# Cooperative Phase Adaptation and Amplitude Amplification of Neuronal Activity in the Vagal Complex: An Interplay Between Microcircuits and Macrocircuits

**DOI:** 10.3389/fnsys.2019.00072

**Published:** 2019-12-03

**Authors:** Yoshinori Kawai

**Affiliations:** Department of Anatomy, The Jikei University School of Medicine, Nishi-Shimbashi Minato-ku, Tokyo, Japan

**Keywords:** resonance, synchrony, noise, fluctuation, brain wave, electrophysiology, emergence, complex adaptive system

## Abstract

Clusters of neurons can communicate with others through the cross-frequency coupling mechanism of oscillatory synchrony. We addressed the hypothesis that neuronal networks at various levels from micro- to macrocircuits implement this communication strategy. An abundance of local recurrent axons of vagal complex (VC) cells establish dense local microcircuits and seem to generate high-frequency noise-causing stochastic resonance (reverberation) and coherence resonance, even in *in vitro* slice preparations. These phenomena were observed *in vitro* as the generation of episodes of higher-frequency noise after an external stimulation and as stimulus-induced or spontaneous high-amplitude signals (postsynaptic activities). The *in vitro* microcircuit networks rarely sustained the stochastic resonance and coherence resonance cooperatively; however, *in vivo* networks involving additional intrabulbar mesocircuits and large-scale macrocircuits were able to sustain them cooperatively. This gave rise to large-scale oscillatory synchrony leading to robust power and coherence of signals with high amplitudes, reaching several millivolts in amplitude from a noise level of ~100 microV through cardiorespiratory frequency coupling. A regenerative mechanism of neuronal circuits might work for the generation of large-scale oscillatory synchrony. The amplitude and phase of neuronal activity *in vivo* may interact cooperatively to give rise to varying degrees of power and coherence of robust rhythmic activity for distinct physiological roles. The cooperative interaction between phase adaptation and amplitude amplification of neuronal activity may provide diverse nervous systems with both robustness and resilience.

## Introduction

The most salient feature of brain electrical activity is the oscillatory synchrony generated and/or sustained by ensembles of coupled neuronal oscillators (Destexhe et al., 1999; Buzsaki, 2006; Canolty and Knight, 2010; Kawai, 2018b). Clusters of neurons with varying spatial dimensions and connectivity form regenerative neuronal circuits that can elicit synchronized oscillations. These neuronal circuits also incessantly generate brain activity at the level of noise in the form of local sub- and suprathreshold waves, in addition to spatially distributed large-scale oscillatory synchrony. Previous publications have addressed and emphasized a possible interdependent relationship between wave synchrony and noise, although rarely in real nervous tissues (but see Galán et al., 2006), and mainly in theoretical or simulation-based studies (Ermentrout et al., 2008; Faisal et al., 2008). For example, it has been reported that noise can play a stabilizing role in synchronized oscillations. When adequate random noise is added, stable and synchronized oscillations may appear. Uncorrelated noise may thus sufficiently change the characteristics of a non-oscillating feedback system to produce stable oscillations (Springer and Paulsson, 2006). Theoretical and simulation-based studies predict that stochastic noise or fluctuation in an excitable-system can produce large-scale oscillatory synchrony *via* stochastic or coherence resonance mechanisms (Wiesenfeld and Moss, 1995; Pikovsky and Kurths, 1997; McDonnell and Abbott, 2009; Dodla and Wilson, 2010).

However, noise-based stochastic or coherence resonance-like phenomena have rarely been described with respect to the nervous system, particularly not in real *in vitro* and *in vivo* brain preparations that retain neuronal circuits with varying levels of intactness (Galán et al., 2006). Using brainstem preparations, the present study sought to investigate the structure and dynamics of neuronal activity [subthreshold, spike, local field potential (LFP) activity] at the noise level that may develop into emergent large-scale oscillatory synchrony. In addition, the possible functional significance of such developmental dynamics was evaluated (Kawai, 2018b). Although the significance of noise in neural functions has been both endorsed and refuted in previously published literature (Stein et al., 2005; Ermentrout et al., 2008), the present study would like to stress the quintessential role of neuronal noise in neural functions. Analogous to complex adaptive systems, a cooperative interaction of wave amplitude amplification and phase adaptation is proposed in the present study with respect to the robustness and resilience of these systems (Holland, 1995).

## Materials and Methods

### Animal Preparations and Electrophysiological Recordings

All surgical and experimental procedures were approved by the Institutional Committee for the Care and Use of Experimental Animals at the Jikei University School of Medicine in Japan and were performed in accordance with the Guidelines for Proper Conduct of the Animal Experiments by the Science Council of Japan.

For *in vitro* preparations, Sprague–Dawley rats (postnatal days 18–24; Saitama Experimental Animals Supply, Japan) were deeply anesthetized with ether. After decapitation at the cervical spinal level followed by rapid craniotomy, the brainstem mass including the cerebellum was quickly removed, and a 2–3-mm-thick block containing the area postrema (caudal medulla oblongata) was prepared for coronal slicing. Two slices containing the area postrema for patch-clamp whole-cell recordings were usually available from each animal.

Coronal slices (250–300 μm thickness) were made using a micro slicer (DTK-1000; Dosaka, Japan). Slices containing the area postrema were collected and incubated in standard Ringer’s solution for at least 1 h at 37°C. The standard Ringer’s solution had the following composition (in mM): 125 NaCl, 2.5 KCl, 2 CaCl_2_, 1 MgCl_2_, 1.25 NaH_2_PO_4_, 26 NaHCO_3_, and 10 glucose. This solution was continuously bubbled with a mixture of 95% O_2_ and 5% CO_2_ (pH 7.4, ~320 mOsm). After the incubation, a single brain slice was transferred to a recording chamber placed on the stage of an upright microscope (BX51WI; Olympus, Japan) and submerged in the continuously superfusing medium (1–2 ml/min). Whole-cell recordings with a high seal resistance (>1 GΩ before break-in) were obtained from cells of the nucleus of the tractus solitarius (NTS) using borosilicate glass pipettes [1.5 mm outer diameter (O.D.); World Precision Instruments, Sarasota, FL, USA]. The electrodes contained (in mM): 140 cesium-acetate, 0.1 CaCl_2_, 2 MgCl_2_, 5 TEA, 1 EGTA, 10 HEPES, 5 ATP, and 0.1% biocytin (pH 7.3). Unless specified otherwise, drugs were purchased from Sigma-Aldrich (St. Louis, MO, USA). The resistance of the electrodes filled with this solution ranged from 5 to 12 MΩ. Neuronal signals were recorded in either the voltage-clamp or the current-clamp mode (Multiclamp 700A; Molecular Devices, Foster City, CA, USA). Signals were filtered at 1–2 kHz and digitized at 2–4 kHz.

For detecting inward excitatory postsynaptic currents (EPSCs) and outward inhibitory PSCs (IPSCs) in the same neurons, the membrane potential was clamped first between −60 and −70 mV and then between 0 and 10 mV, respectively. Upward and downward currents of peak amplitudes more than twice the device noise level (~20 pA) were sampled.

Evoked EPSCs and IPSCs were also recorded and analyzed. Isolated stimuli of 100–200 μs duration were applied at ~0.1 Hz through tungsten bipolar electrodes with a tip diameter of 20 μm and separation of 150 μm. The electrodes were positioned at the dorsomedial part of the tractus solitarius in coronal slices.

On occasion, the A-type γ-aminobutyric acid (GABA_A_) receptor antagonist bicuculline methiodide (10 μM) and the non- N-methyl-D-aspartate (NMDA) glutamate receptor antagonist 6-cyano-7-nitroquinoxaline-2,3-dione disodium (10 μM) were bath-applied to isolate excitatory glutamate- and inhibitory GABA-mediated activities, respectively.

For *in vivo* preparations, electrophysiological recordings were carried out using five male Sprague–Dawley rats (weight range, 280–310 g). Animals were anesthetized with an intraperitoneal injection of ketamine (30 mg/kg) and xylazine (24 mg/kg) and placed in a stereotaxic instrument for recording. In most cases, 0.5% isoflurane was additionally administered through a nose mask to obtain sufficient depth of anesthesia during recordings.

Glass electrodes (1.5 mm O.D.; World Precision Instruments) containing 2 M NaCl were used in *in vivo* extracellular recordings. The resistance of the electrodes filled with this solution ranged from 1 to 5 MΩ. After making an incision in the atlanto-occipital dural membrane, an electrode tip was advanced under a stereoscopic microscope vertically with a motorized micromanipulator (IVM Single; Scientifica, East Sussex, UK) into the exposed left dorsal medulla at the level of the area postrema; the depth was 50–500 μm from the brain surface. Neuronal signals were recorded in alternating current mode with a Multiclamp700A. The amplified signals were analyzed offline using Spike2 (Cambridge Electronic Design Limited, Cambridge, UK) and OriginPro2017 (Lightstone Company, Tokyo, Japan) software.

Simultaneous 16-channel *in vivo* recordings were performed from the vagal complex (VC) using a silicon probe (A1x16-Poly2s-5mm-50s-177-A16; NeuroNexus Technologies Inc., Ann Arbor, MI, USA). The resistance of each electrode specified by the manufacturer was between 0.96 and 1.17 MΩ. Each electrode “site” consisted of a circular platinum metal 15 μm in diameter, arranged by two 8-site-columns, and separated by 50 μm (Blanche et al., 2005). Electrical activities were amplified (A-M Systems Model 3600 Amplifier; Carlsborg, WA, USA), sampled at 1–4 kHz, and stored for offline analysis.

Cardiorespiratory activities were recorded non-invasively with a piezoelectric pulse transducer (PZT; MP100; AD Instruments, New South Wales, NSW, Australia). The PZT transformed the mechanical movement or vibration of the thorax (through touch on the sensor probe patch) into electrical signals that could be divided into heartbeat and respiration components (Sato et al., 2006).

### Data Analysis

**Figure 1:**
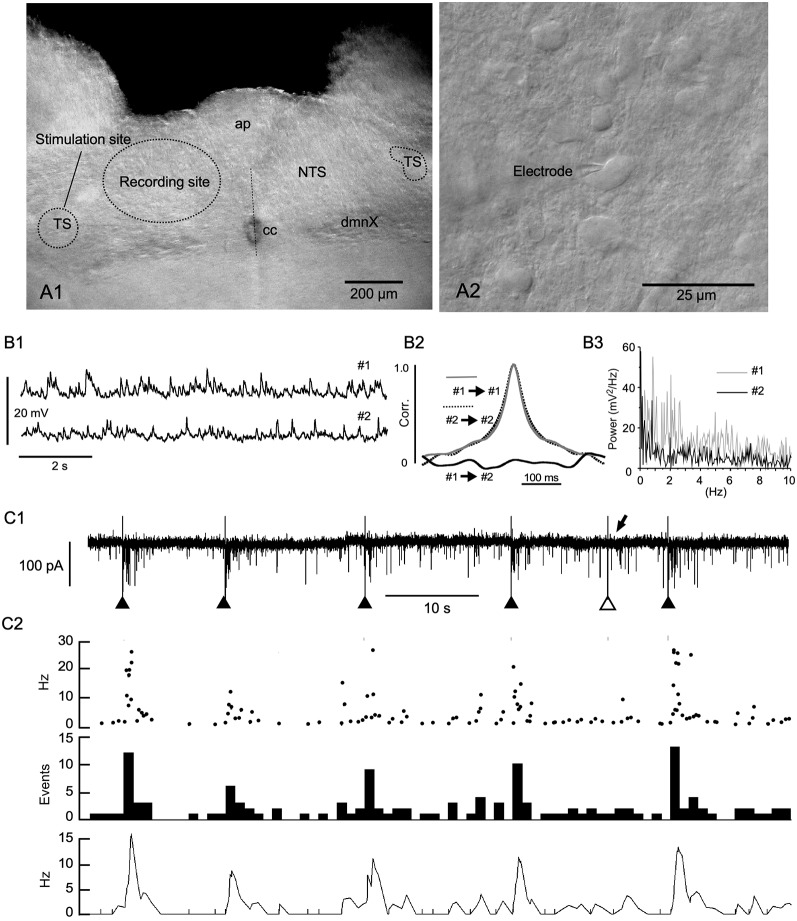
Evoked phase adaptation of postsynaptic activities *in vitro*. **(A1)** A differential interference contrast (DIC) photomicrograph showing the recording and stimulation sites for *in vitro* slice preparations. **(A2)** A DIC photo of higher magnification. An electrode tip is shown. Note the size and density of NTS cells. **(B1)** Postsynaptic potentials (PSPs) recorded from two nearby neurons (#1 and #2) in a slice preparation using the patch-clamp whole-cell technique in the current-clamp mode. **(B2)** Cross- and auto-correlations (Corr.) of the PSPs. The lack of correlation between the PSP activities of the two cells shows that most of these spontaneous PSP activities were noise or fluctuation. **(B3)** Power spectra (0–10 Hz range) of the PSPs (#1 and #2) of 20 s duration. No specific frequency peaks or spectra coincidences between the PSPs are noted, characterizing the PSP activity features as noise. **(C1,2)** The frequency (phase) of noise activities recorded as postsynaptic currents (PSCs) is increased transiently over several seconds after an external stimulation of input fibers (triangles in **C1**). Of six successive stimulations, one (the open triangle in **C1**) fails to elicit a sufficient increase in frequency (arrow in **C1**). The phase adaptation elicited by external inputs are characterized by instantaneous (Inst in **C2**) frequencies of more than 10 Hz, increased mean (Mean in **C2**) events, and rate (Rate in **C2**) frequencies following subthreshold stimulations (gray vertical lines). ap, area postrema; cc, central canal; dmnX, the dorsal motor nucleus of the vagus; NTS, nucleus of the tractus solitarius; TS, tractus solitarius.

Event data displays (Figure 1) were made using Spike2 menu commands. “Instantaneous frequency (Inst)” takes the inverse of the time difference between the current event and the one preceding it. The event is plotted as a dot. The x-axis dot position is the time event. The y-axis dot position is the instantaneous frequency of that event in Hz with respect to the previous event. “Mean frequency (Mean)” is calculated over the preceding data at each event. “Rate” counts how many events fall within a time period (1 s) and displays the result in the form of a histogram.

Neuronal signals recorded *in vivo* exhibited, to a highly variable degree, a mixture of single- or multi-unit spikes and LFPs, especially when using standard glass electrodes, whereas signals recorded with a silicon probe mostly consisted of LFPs. For 0–10 Hz phase (frequency range of cardiorespiratory rhythms) enhancement, neuronal signals were, in some cases, offline filtered with a low-pass type II Chebyshev filter (Spike2, low-filtered between D.C. and 100 Hz with an order of 2 and a ripple of 60).

Amplitude amplification of *in vitro* spontaneous EPSCs, *in vivo* multiple unit activities (MUAs), and *in vivo* LFPs was evaluated in terms of their height change and signal interval. Records of spontaneous signals (each ~40-s duration) were sampled from each recording mode. The height of the signal amplification was expressed as a mean signal/noise ratio obtained from several experimental sessions (number: 4–8). Intervals between successive pairs of the amplified signals were measured and expressed as frequency (Hz). Values were expressed as mean ± standard errors.

Cross- and auto-correlograms, as well as fast Fourier transform power spectra, were generated with OriginPro2017. Continuous wavelet transform (CWT) and wavelet coherence using Morse wavelets (default wavelet function) were calculated with MATLAB (The MathWorks, Natick, MA, USA). CWT and wavelet coherence were expressed as time-resolved power and coherence spectra, respectively. A detailed explanation of each formula for numerical analysis was previously provided (Kawai, 2018b).

## Results

The caudal parts of the NTS consist of small, densely packed cells which are densely innervated by recurrent local axons that establish both excitatory and inhibitory synaptic transmission (Figures 1A1,A2, Negishi and Kawai, 2011). This microcircuit configuration of the caudal NTS seems to constitute an extremely noisy environment. The noise consisted mostly of subthreshold high-frequency postsynaptic activity even in *in vitro* slice preparations (Figure 1B1). The frequency of excitatory inward currents recorded from a small cell in the whole-cell mode of the patch-clamp technique was between 1.1 and 18.0 Hz (6.2 ± 1.2 Hz, *n* = 12). The postsynaptic activity was considered to be noise because paired activity recorded simultaneously from two neighboring cells (less than 50 μm in distance) showed no correlation or any specific corresponding spectra (Figures 1B2,B3). However, this noisy environment of the NTS rarely developed into a persistent firing even in *in vivo* experiments.

Phase adaptation described in this study represents an increase in the frequency of signal or noise associated with spontaneous or evoked signals of an amplified amplitude. In* in vitro* preparations, subthreshold EPSCs were analyzed because the spontaneous occurrence of spikes was extremely rare in the VC, while spikes were easily evoked after a slight external stimulation of afferent synapses. In contrast, neuronal signal or noise recorded *in vivo* as MUAs and LPFs would consist mostly of spike activity. A possible cooperative relationship between amplitude amplification and phase adaptation was addressed using both *in vitro* and *in vivo* preparations.

### Evoked Phase Adaptation of Postsynaptic Activities *in vitro*

Evoked EPSCs were recorded from small NTS cells by minimally stimulating the tractus solitarius (Figure 1C1). Each tractus solitarius stimulation elicited usually a monosynaptic EPSC that possibly derived from primary afferents or nearby cells or polysynaptic EPSCs that could last for up to several seconds (solid triangles in Figures 1C1, 2). In a few cases, the tractus solitarius stimulation failed to generate apparent polysynaptic EPSCs (open triangle and arrow in Figure 1C1). An evoked elevation of secondary polysynaptic activities was inconsistently observed (Figure 1C2). This statistically significant increase in noise frequency after an external stimulation was indicative of “reverberation of recurrent activity” (Tegnér et al., 2002). The reverberation reached 20–30 Hz in the instantaneous frequency (Inst in Figure 1C2), more than 10 Hz in the mean frequency (Mean in Figure 1C2), 10–15 Hz in the rate count (Rate in Figure 1C2) and lasted for 1–5 s (Figure 1C2).

**Figure 2:**
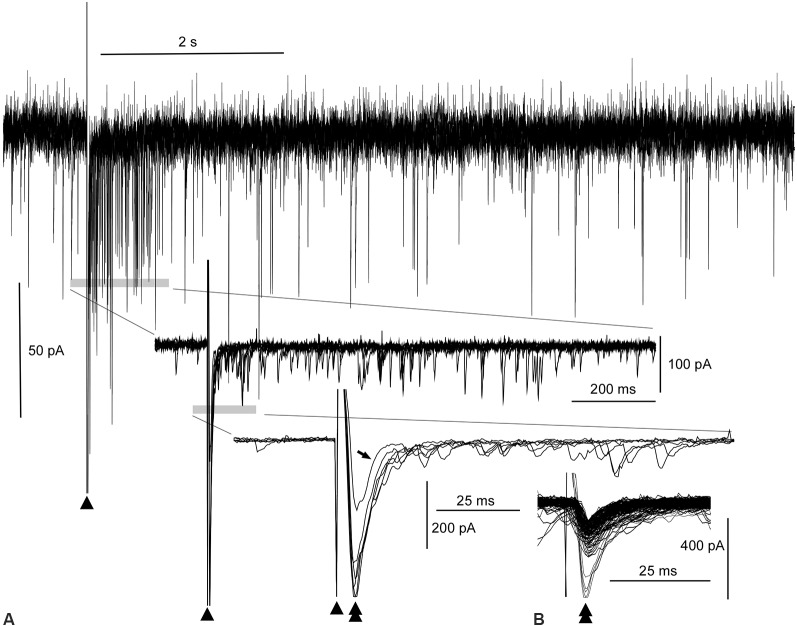
Evoked amplitude amplification of postsynaptic activities *in vitro*. **(A)** Superimposition of six episodes of PSCs following external stimulations (arrowheads), as indicated in Figure 1. During the 1-s period after a stimulation, an apparent increase in the frequency of inward PSCs is noted (gray bar in the top wave trace). Both upward and downward stimulus artifacts are truncated in the top wave trace. Traces (middle and bottom) further expanded near the stimulus artifacts reveal high amplitude inward (downward) PSCs (double arrowhead in the bottom trace) following the stimulus artifacts (arrowheads). Out of six PSCs following the stimuli, one PSC of low amplitude fails to give rise to an increase in frequency (arrow in the bottom trace, see also the open arrowhead in Figure 1C). In the middle and bottom wave traces, upward traces near the stimulus artifacts are truncated. Note the co-occurrence of preceding high-amplitude PSCs and episodes of high-frequency PSCs for an increase in frequency. **(B)** Inward trough (double arrowheads)-triggered superimposition of both spontaneous and evoked PSCs. The inward evoked PSC with an aborted increase in frequency (tilted arrow, bottom wave trace in **A**) is embedded in many other spontaneous PSCs whose amplitudes are all within the noise level.

### Evoked Amplitude Amplification of Postsynaptic Activities *in vitro*

The superimposition of a preceding evoked monosynaptic EPSC and the following multiple polysynaptic EPSCs by adjusting the stimulus artifacts (triangles in Figure 1C1; *n* = 6) was utilized to better assess the relationship between them (Figure 2A). A reverberation is most evident within ~1 s following the stimulation (top wave trace, Figure 2A). More expanded wave traces (middle and lower traces, Figure 2A) indicate that multiple polysynaptic EPSCs appear more frequently near the preceding evoked EPSCs (double arrowheads in the lower expanded wave traces, Figure 2A). However, a monosynaptic EPSC of lower amplitude (206 pA) fails to generate a barrage of subsequent multiple polysynaptic EPSCs (arrow in the lower group of traces, Figure 2A; see also Figure 1C1). It seems that the preceding evoked monosynaptic EPSCs with an amplitude (423 ± 42.1 pA; *n* = 5) large enough to elicit a reverberation cause the following multiple polysynaptic EPSCs, and thus amplitude amplification and phase adaptation co-occur by external stimulation. Superimposition of spontaneous and evoked EPSCs according to their inward current troughs (Figure 2B) indicates that the amplitude of an evoked EPSC failing to elicit a reverberation was within the range (40–260 pA) of that of spontaneous EPSCs, i.e., of a fluctuation (noise) level.

### Spontaneous Amplitude Amplification of Electrical Activities *in vitro* and *in vivo*

**Figure 3:**
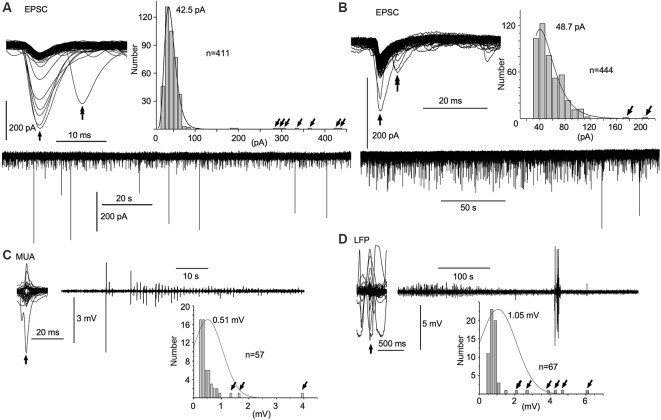
Spontaneous amplitude amplification of electrical activities *in vitro* and *in vivo*. **(A,B)** Two examples of PSCs recorded in slice preparations using the patch-clamp whole-cell technique in the voltage-clamp mode. Inward trough (arrows)-triggered PSCs are superimposed. Several inward PSCs (arrows) are followed by late PSCs (double arrows). Note that high-amplitude PSCs occur randomly with an extremely low frequency in the PSC amplitude histograms (tilted arrows) and the original continuous PSC recordings. Note, the average of PSC amplitudes is 40–50 pA sampled from more than 400 PSCs. **(C)** Inward trough (arrow)-triggered multiple unit activities (MUAs) recorded *in vivo* with a standard glass electrode are superimposed on an original continuous MUA recording. **(D)** Inward trough (arrow)-triggered local field potentials (LFPs) recorded *in vivo* with a silicon electrode are superimposed on an original continuous LFP recording. Note that high-amplitude signals occur sporadically with higher frequency in the amplitude histograms (tilted arrows). In *in vivo* recordings, episodes of repetitive high-amplitude activities over several tens of seconds are spontaneously generated and subdued. EPSC, excitatory postsynaptic current.

The mean amplitude of spontaneous EPSCs in patch-clamp whole-cell recordings of the voltage-clamp mode was 40–50 pA in slice preparations (Figures 3A,B). The distribution of current amplitudes revealed a logarithmic normal distribution with a long tail. A spontaneous amplitude amplification rarely occurred in less than 2% of EPSCs. The spontaneous EPSCs superimposed with their inward current troughs indicate that several preceding EPSCs are followed by late EPSCs (5–10 ms latency) with a hint of spontaneous phase adaptation, however, on a few rare occasions, if any at all.

In contrast to *in vitro* preparations, a wave amplitude amplification was seen more frequently and conspicuously *in vivo* in terms of occurrence frequency (5–10%) and amplitude amplification (Figures 3C,D). Neuronal activities in the NTS recorded with a standard glass electrode comprised mostly a mixture of single- and multi-unit spikes, as well as highly polyphasic and LFP-like longer duration waves of up to several 100 μV in amplitude (i.e., the noise level). In a few cases, neuronal activities with an amplified amplitude (>1 mV) emerged abruptly, ensued sporadically for several seconds, and waned (Figure 3C). Similar phenomena of amplitude amplification were confirmed with LFPs recorded using a silicon electrode (Figure 3D). Spontaneous amplitude amplification of neuronal activities was observed in the NTS of both *in vitro* and *in vivo* preparations.

### Cooperative Phase Adaptation and Amplitude Amplification of Spontaneous Neuronal Activities *in vivo*

**Figure 4:**
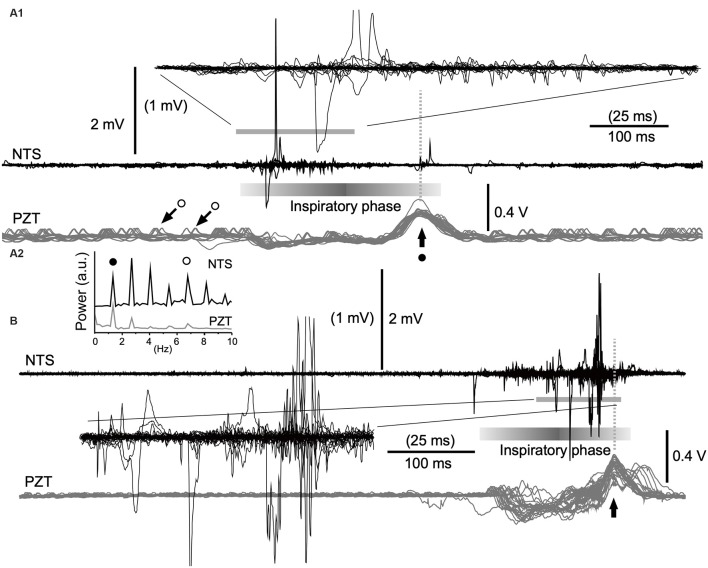
Cooperative phase adaptation and amplitude amplification of neuronal activities *in vivo*. **(A1,B)** Two examples showing high-amplitude neuronal electrical activities recorded with a standard glass electrode. The majority of high-amplitude signals are synchronized with the inspiratory phase according to simultaneously recorded signals (gray traces) of a piezoelectric transducer (PZT) attached to the thorax. The superimposition according to PZT upward peaks (vertical arrows) of simultaneous neuronal (NTS) and PZT recordings shows several tens of consecutive episodes of one respiratory cycle (**A1**, 30 cycles; **B**, 26 cycles). Expanded wave traces are truncated and scaled to values in parentheses **(A1,B)**. **(A2)** Power spectra of neuronal (NTS) and cardiorespiratory (PZT) rhythms. Note synchrony of these two waves. Solid and open circles indicate respiratory and cardiac fundamental frequencies (~1.3 Hz and ~6.8 Hz, respectively). They are represented respectively as large (solid circle with arrow) and small (open circles with arrows) upward waves in the PZT trace (**A1**). NTS, nucleus of the tractus solitarius; a.u., arbitrary unit.

In *in vivo* preparations, it seemed that amplitude amplification occurred in concert with the respiratory rhythm (Kawai, 2018b). In order to verify this in more detail, cardiorespiratory and neuronal NTS activities were simultaneously recorded using a non-invasive PZT placed under the thorax (Figures 4A1,B). The power spectra of neuronal (NTS in Figure 4A2) and cardiorespiratory (PZT in Figure 4A2) rhythms show clear coherence. The respiratory and cardiac fundamental frequencies were ~1.3 Hz and ~6.8 Hz, respectively. Superimposition of simultaneous neuronal and PZT activities triggered by each large peak of PZT activity (that roughly corresponds to a transition from the inspiratory to the expiratory phase) indicates that amplitude amplification and higher frequency of noise co-occur during each inspiratory phase (Figures 4A1,B). However, strikingly large amplitudes of neuronal activity are concentrated either in the initial (Figure 4A1) or the final (Figure 4B) segment of an inspiratory phase.

### Emergence of Large-Scale Cooperative Phase Adaptation and Amplitude Amplification of Neuronal Activities *in vivo*

**Figure 5:**
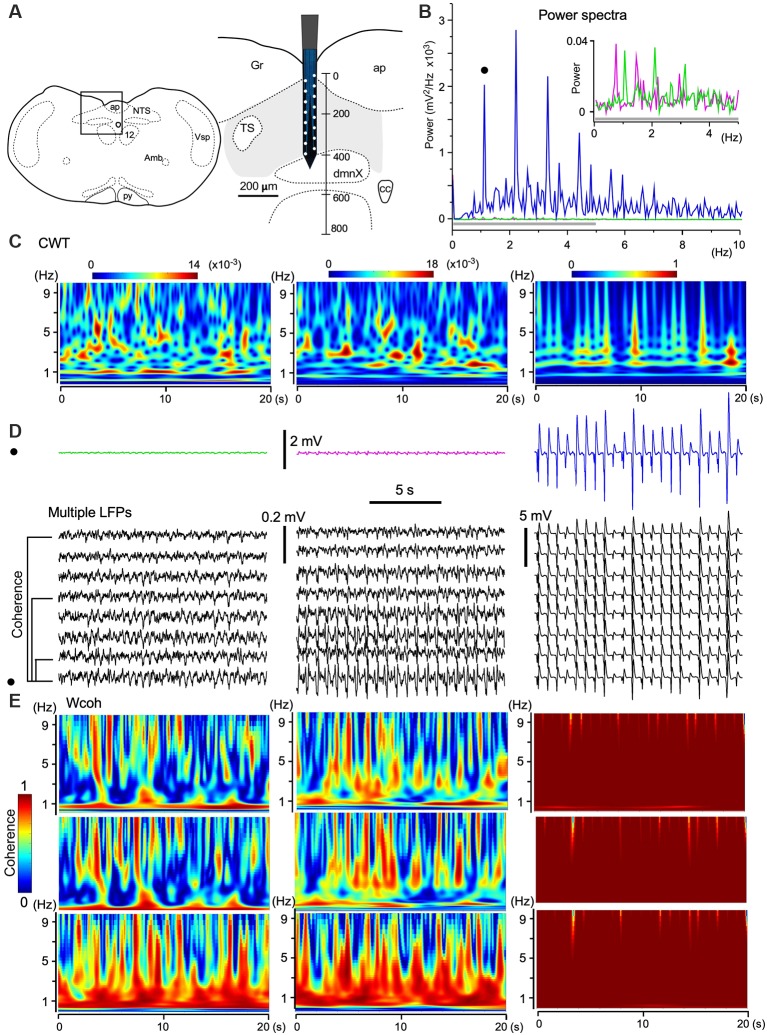
The emergence of large-scale cooperative phase adaptation and amplitude amplification of neuronal activities *in vivo*. **(A)** A silicon electrode records LFPs at multiple sites over a 100 μm along the depth of the vagal complex (VC). Right, Magnification of the picture from an anatomical atlas (left). **(B,C)** Twenty-second **(B)** and time-resolved (**C**; continuous wavelet transform, CWT) power spectra of three successive episodes (20 s) of LFPs at a certain fixed recording site (three traces in **D**, top). **(B)** The power of high-amplitude LFPs (in blue) is increased compared to those of low-amplitude LFPs (in magenta and green, inset for a gray bar region). This representative set of episodes (solid circle in **D**) is one of eight sets of traces (**D**, multiple LFPs). **(E)** Time-resolved coherence spectra (wavelet coherence, Wcoh) between pairs of traces of the representative episode (solid circle in **D**) and of those at different distances (50, 200, and 350 μm apart, **D**, bottom). Note that the shorter the distance of paired sites or the larger the amplitude of paired waves is, the larger the coherence is. Amb, ambiguous nucleus; ap, area postrema; cc, central canal; dmnX, the dorsal motor nucleus of the vagus; Gr, gracilis nucleus; NTS, nucleus of the tractus solitarius; nXII (12), hypoglossal nucleus; py, pyramidal tract; TS, tractus solitarius; Vsp, spinal nucleus of the trigeminal nerves.

Given that each NTS cell fires synchronously during an inspiratory phase, large-scale oscillatory synchrony was expected to be recorded in the VC with silicon multielectrode. Since the silicon electrode had a large vertical dimension of ~400 μm, the part of the medulla oblongata termed the VC, consisting of the caudal NTS and the dorsal motor nucleus of the vagus nerve, was used instead of the NTS, to improve precision (Figure 5A).

As shown in Figure 3D, the amplitudes of LFPs changed sporadically. To quantify the three different phases of wave activities, 20-s (Figure 5B) and time-resolved (Figure 5C; CWT) power spectra were applied to three successive episodes (in magenta, green, and blue; 20 s durations, Figure 5D) of LFPs at a certain fixed recording site of the electrode. The power spectra show that the larger the signal amplitude the larger the power and that stronger power signals converge to a 1–3 Hz frequency (delta) band (Figure 5C). Time-resolved coherence spectra (wavelet coherence, Wcoh) between trace pairs with different distances (50, 200, and 350 μm) indicated that the shorter the distance of two paired recording sites or the larger the amplitude of the paired waves is, larger is the coherence between those wave pairs (Figure 5E). A large amplitude with a large-scale phase adaptation of LFPs generated larger power and coherence over a larger brain area.

### Quantitation of Amplitude Amplification and Frequency of Spontaneous Signals

**Figure 6:**
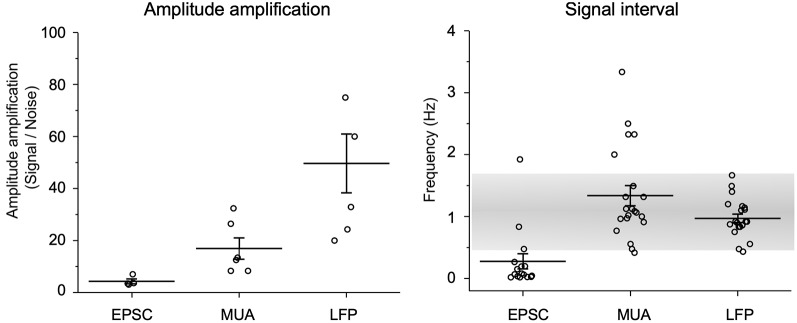
Quantification of amplitude amplification and frequency. Amplitude amplification of *in vitro* spontaneous EPSCs, *in vivo* MUAs, and *in vivo* LFPs are quantified according to height changes and signal intervals. Note that the frequency of amplified signals in *in vivo* recordings adapts to a range of respiratory rhythmic cycles (~1 Hz: gray shaded range). Values are represented as mean ± standard errors.

Amplitude amplification of *in vitro* spontaneous EPSCs, *in vivo* MUAs, and *in vivo* LFPs was evaluated according to the height changes and signal intervals (Figure 6). Values for amplitude amplification of spontaneous EPSCs, MUAs, and LFPs were 4.3 ± 0.9 (*n* = 4, each session contained 15 samples), 18.6 ± 4.6 (*n* = 5, 17 samples), and 49.6 ± 11.3 (*n* = 8, 18 samples), respectively. Values for the frequency of the amplified signals were 0.28 ± 0.12 (*n* = 15), 1.34 ± 0.16 (*n* = 21), and 0.97 ± 0.07 (*n* = 21), respectively. The frequency of amplified signals in *in vivo* recordings was adapted to a range of respiratory rhythmic cycles (~1 Hz).

## Discussion

Large-scale oscillatory synchrony emerges spontaneously in the VC of anesthetized animals (Kawai, 2018b). Neuronal activity changes incessantly in frequency and amplitude depending on the spatial dimension of the oscillatory synchrony (Destexhe et al., 1999; Buzsaki, 2006). This phenomenon implies that the important properties of neuronal activity, i.e., phase and amplitude, may interact interdependently. The cooperative dynamics of wave amplitude and phase were addressed in the present study with respect to neuronal circuit configuration or dimension.

### Microcircuits and Macrocircuits Involving the Vagal Complex

The VC consists of the caudal NTS and the dorsal motor nucleus of the vagus nerve (Ramon y Cajal, 1995; Kawai, 2018a,b). The caudal NTS provides multiple connections with diverse brain areas encompassing the telencephalon to the spinal cord, thus establishing large-scale macrocircuits (Kawai, 2018a). Most brain areas innervated by the NTS establish reciprocal connections with the NTS and form regenerative macrocircuits including intrabulbar brainstem mesocircuits that govern a robust rhythmic cardiorespiratory activity. This multiple-nested circuit configuration may implement noise-based stochastic synchrony that could confer a benefit to such a system in which robust cardiorespiratory rhythmicity and resilience to external perturbation coexist cooperatively. In addition to macrocircuits, the NTS contains microcircuits in which dense recurrent axons generate a highly noisy neuronal activity (Negishi and Kawai, 2011). The noise-based synchrony of cooperative wave phase adaptation and amplitude amplification in NTS microcircuits is specifically attributed to their cytoarchitectural features. The NTS consists of an extremely concentrated assembly of synaptically interconnected small cells (~11 μm in diameter; Yoshioka et al., 2006). This structural compactness enables a clearer recording of emergent noise-based synchrony in *in vivo* preparations with a typical glass electrode, since the detection of a wave amplitude amplification is relatively easy with a stochastic correlation of noise activity. This may be due to the fact that a given receptive electrical field at an electrode tip would contain much greater numbers of smaller cell soma. This seems to provide a functional significance for sensory processing in reference to intrinsic stochastic synchrony since the NTS is strategically the sole recipient of peripheral viscerosensory information while connecting central macro-circuits governing rhythmic cardiorespiratory activity. This cooperative interplay between microcircuits and macrocircuits would be of functional significance.

Different behaviors of phase adaptation and amplitude amplification in *in vitro* and *in vivo* preparations could result from a developmental change in circuits rather than the different dimensions (micro vs. macro) of matured circuit organization. However, it has been demonstrated anatomically and physiologically that the VC circuit matures until late in the third week of postnatal development, supporting the latter possibility (Yoshioka et al., 2006; Tashiro and Kawai, 2007).

### Neuronal Activity as Noise and Signal

The hierarchical architecture of nested neuronal circuits involving the VC could provide the anatomical basis for its unique task for VC for viscerosensory information processing (the caudal NTS) and autonomic output production (the dorsal motor nucleus of the vagus nerve) in addition to centrally generated neuronal activity. The centrally generated neuronal activity consists mostly of spontaneous stochastic noise that can change into signals of varied spatiotemporal dimensions and dynamics based on the cross-frequency coupling of the cardiorespiratory frequency range (Kawai, 2018b).

Wave synchrony and oscillation are, in most cases, phenomena that co-occur during neuronal activity, but the relative proportion of power in a certain macroscopic phenomenon varies according to the required task of the neuronal activity (Destexhe et al., 1999; Buzsaki, 2006). In the large-scale neuronal activity, wave synchrony with large amplitudes would be more appropriate for a signal transfer over longer distances to multiple destinations. For local activities, wave oscillations with a fine-tuned phase (particularly those of the higher gamma frequency range) would be appropriate for holding more precise information. The fundamental feature of ongoing neuronal activity is stochastic fluctuation (noise) to enable a potential development into a signal in either direction according to the changing environment to which individuals must adapt.

Gap junctions between neurons may play an important role in synchronized rhythms (Konopacki et al., 2014). However, this is unlikely to be the case in the VC because intracellular injections of biocytin or lucifer yellow, which can penetrate the junction complex, were not reported to stain any neighboring cells (Yoshioka et al., 2006; Negishi and Kawai, 2011).

### Emergence and Development of Stochastic Synchrony

The phenomenon described in the present study seems to be similar to stochastic synchrony investigated in the olfactory bulb (Galán et al., 2006) in that both are likely to be noise-induced synchronization. Correlated noisy inputs are able to generate synchronous oscillation of the gamma frequency range (~40 Hz) in mitral cells of the olfactory bulb *in vitro*. Of note is the clear difference in frequency ranges between low (delta–theta for the VC, present study) and high (gamma for olfactory mitral cells; Galán et al., 2006). The stochastic synchrony emerges due to the influence of partially correlated but aperiodic transient inputs; neither synaptic coupling nor oscillatory input is required. In this respect, this phenomenon should be designated as coherence resonance (Pikovsky and Kurths, 1997) but not stochastic resonance (McDonnell and Abbott, 2009). Stochastic resonance has been used to explain noise-dependent entrainment of neuronal firing to a subthreshold oscillatory input in a variety of systems (Wiesenfeld and Moss, 1995; McDonnell and Abbott, 2009), including crayfish mechanoreceptors (Douglass et al., 1993; Moss and Pei, 1995). Although the adaptive feature of spiking synchrony and periodic network bursts was also investigated in neuronal networks (Mainen et al., 1995; Fardet et al., 2018), a relevance to stochastic noise was not addressed. The spontaneous synchrony, in this case, seems to be generated by phase-adaptive ion channel properties rather than stochastic noise.

### Complex Adaptive System

The term complex adaptive system states that complex, emergent, and macroscopic properties of the system as a whole (an ensemble) could be self-organized as a result of non-linear dynamics of interacting microscopic elements, where they have no *a priori* plan or meaning (Holland, 1995; Kelso, 2016). The system is also characterized by a high degree of adaptive capacity (adaptation or homeostasis), giving it resilience in the face of perturbation. The microscopic interactions are non-linear, such that small changes in inputs, physical interactions, or stimuli can cause large effects or significant changes in outputs. Any interaction can feedback onto itself directly or after a number of intervening stages. Such feedback can vary in quality. This interaction may be designated as regenerative recurrence or iteration. The overall behavior of the system of elements would not be predicted by the behavior of the individual elements.

The above-mentioned description concerning a complex adaptive system may be applicable to many aspects of stochastic synchrony of the VC neuronal activity revealed in this study. The results show that a stable frequency of robust neuronal activity ranging to respiration rhythms emerges *in vivo* networks and would adapt to a changing environment.

## Conclusion

The maintenance of rhythmic cardiorespiratory brain activity, which is the most fundamental and robust activity, may be a prerequisite for sustaining life. This robust task is attributed essentially to neuronal networks of the brainstem responsible for rhythmic cardiorespiratory activity (Feldman and Ellenberger, 1988). This task also requires resilience in the face of immediate changes in the environment, which individuals must constantly adapt to (Dick et al., 2014). The activity of the brainstem network involving the VC must obey a system rule in which robustness and resilience cooperatively and dynamically coexist. The most promising candidate for a system model may be that of a complex adaptive system (Holland, 1995; Kelso, 2016). This system contains concepts with several important keywords, including but not limited to robustness and resilience, self-organization, synchrony, non-linear dynamics, and emergence. Studies of system dynamics addressing such perspectives warrant multidisciplinary investigations using both experimental and theoretical approaches.

## Data Availability Statement

All datasets generated for this study are included in the article.

## Ethics Statement

The animal study was reviewed and approved by the Care and Use of Experimental Animals at the Jikei University School of Medicine in Japan.

## Author Contributions

YK planned and performed the experiments, analyzed the results and wrote the manuscript.

## Conflict of Interest

The author declares that the research was conducted in the absence of any commercial or financial relationships that could be construed as a potential conflict of interest.
